# Global proteomic profiling of multiple organs of cats (*Felis catus*) and proteome-transcriptome correlation during acute *Toxoplasma gondii* infection

**DOI:** 10.1186/s40249-022-01022-7

**Published:** 2022-09-14

**Authors:** Lan-Bi Nie, Wei Cong, Jun-Jun He, Wen-Bin Zheng, Xing-Quan Zhu

**Affiliations:** 1 College of Veterinary Medicine, Shanxi Agricultural University, Taigu, Shanxi 030801 People’s Republic of China; 2 State Key Laboratory of Veterinary Etiological Biology, Key Laboratory of Veterinary Parasitology of Gansu Province, Lanzhou Veterinary Research Institute, Chinese Academy of Agricultural Sciences, Lanzhou, Gansu 730046 People’s Republic of China; 3 College of Animal Science and Technology, Jilin Agricultural University, Changchun, Jilin 130118 People’s Republic of China; 4 Marine College, Shandong University, Weihai, Shandong 264209 People’s Republic of China; 5 Key Laboratory of Veterinary Public Health of Yunnan Province, College of Veterinary Medicine, Yunnan Agricultural University, Kunming, Yunnan 650201 People’s Republic of China

**Keywords:** *Toxoplasma gondii*, Proteomics, Transcriptomics, Cat, mRNA-protein correlation

## Abstract

**Background:**

*Toxoplasma gondii* is a protozoan parasite which can infect almost all warm-blooded animals and humans. Understanding the differential expression of proteins and transcripts associated with *T. gondii* infection in its definitive host (cat) may improve our knowledge of how the parasite manipulates the molecular microenvironment of its definitive host. The aim of this study was to explore the global proteomic alterations in the major organs of cats during acute *T. gondii* infection.

**Methods:**

iTRAQ-based quantitative proteomic profiling was performed on six organs (brain, liver, lung, spleen, heart and small intestine) of cats on day 7 post-infection by cysts of *T. gondii* PRU strain (Genotype II). Mascot software was used to conduct the student’s *t*-test. Proteins with *P* values < 0.05 and fold change > 1.2 or < 0.83 were considered as differentially expressed proteins (DEPs).

**Results:**

A total of 32,657 proteins were identified in the six organs, including 2556 DEPs; of which 1325 were up-regulated and 1231 were down-regulated. The brain, liver, lung, spleen, heart and small intestine exhibited 125 DEPs, 463 DEPs, 255 DEPs, 283 DEPs, 855 DEPs and 575 DEPs, respectively. Gene Ontology (GO) annotation and Kyoto Encyclopedia of Genes and Genomes (KEGG) pathway enrichment analyses of all proteins and DEPs in all organs showed that many proteins were enriched in binding, cell part, cell growth and death, signal transduction, translation, sorting and degradation, extracellular matrix remodeling, tryptophan catabolism, and immune system. Correlations between differentially expressed proteins and transcripts were detected in the liver (*n* = 19), small intestine (*n* = 17), heart (*n* = 9), lung (*n* = 9) and spleen (*n* = 3).

**Conclusions:**

The present study identified 2556 DEPs in six cat tissues on day 7 after infection by *T. gondii* PRU strain, and functional enrichment analyses showed that these DEPs were associated with various cellular and metabolic processes. These findings provide a solid base for further in-depth investigation of the complex proteotranscriptomic reprogramming that mediates the dynamic interplays between *T. gondii* and the different feline tissues.

**Supplementary Information:**

The online version contains supplementary material available at 10.1186/s40249-022-01022-7.

## Background

*Toxoplasma gondii* can infect humans and many animals worldwide [1]. In humans, infection is usually asymptomatic or causes mild flu-like symptoms [1]. However, toxoplasmosis can have serious health effects in immunocompromised patients and cause miscarriage or birth defects in pregnant women [2]. Toxoplasmosis can also have an impact on the animal health and fertility, especially in pigs, resulting in serious economic losses [3, 4].

The cat and other felids serve as the definitive host where they support the formation of *T. gondii* oocysts, which are excreted with feces. *T. gondii* oocysts can contaminate the soil in areas where cats are present, and the oocysts can survive for over a year in non-extreme environments [5–7]. Humans as well as domesticated and wild animals can be infected by consuming water and vegetables containing *T. gondii* oocysts or by ingesting meat containing the parasite tissue cysts [8, 9]. Given the critical role that the cats play in the transmission of *T. gondii*, it is important to enhance our understanding of the pathogenesis of toxoplasmosis in cats. However, many of the mechanisms underlying *T. gondii*-induced molecular and pathological changes in domestic cats remain unknown.

Different technologies have been used to study the molecular attributes of *T. gondii*, and the molecular mechanisms associated with *T. gondii* infection in different hosts, including proteomics [10–12], transcriptomics [13–15], and metabolomics [16–18]. Over the past decade, proteomic approaches have been widely used to improve the understanding of host–pathogen interactions [19, 20]. Proteomics enables a comprehensive high-throughput analysis of proteins in organisms and can reveal the proteomic responses of organisms or tissues to perturbations caused by drug treatments or diseases [21]. A proteomic technique called isobaric tags for relative and absolute quantification (iTRAQ) can provide more reliable quantitative measurements and enable the comparison between samples, compared to proteomic analysis based on two-dimensional differential in-gel electrophoresis [22]. The iTRAQ-based quantitative proteomic technique has been used to study *T. gondii*-host interactions [23, 24]. However, the proteomic response of the different body organs of cats during *T. gondii* infection has yet to be investigated.

In this study, the iTRAQ was used to study the global proteomic alterations in the major body organs of cats during acute *T. gondii* infection. The results showed that *T. gondii* infection affected protein expression and pathways in all examined organs. Additionally, we correlated the proteome data with a transcriptome data of the same organs obtained from the same cats in a previous study [25], to expand our understanding of the differentially expressed transcripts and proteins which play a role in cat–*T. gondii* interactions.

## Methods

### Ethics statement

This study was approved by the Animal Ethics Committee of Lanzhou Veterinary Research Institute, Chinese Academy of Agricultural Sciences (Permit number: LVRIAEC-2014–001). The animals used in the experiments were handled in strict accordance with the Animal Ethics Procedures and Guidelines of the People's Republic of China. Every effort was made to minimize animal distress during the experiment.

### Parasite infection and sample collection

Two litters (*n* = 12) of domestic cats (*Felis catu*s) of the Chinese Lihua breed were purchased from a local breeder. Cats were between 2–3 months of age. All cats were tested serologically and found free of *T. gondii* and the viruses, including feline calicivirus, coronavirus, feline immunodeficiency virus, feline leukemia virus, and feline parvovirus as previously descripted [25]. These cats were divided into four groups, three cats each. Two groups were infected and two groups were used as a control. Cats were housed in a controlled environment on a commercial cat food (Royal Canine Inc., St. Charles, MO, USA). Based on the daily energy needs of individual cats, they were fed individually once per day and water was provided ad libitum.

*T. gondii* PRU strain (Genotype II) maintained in Kunming mice in our laboratory was used in this study [26]. This *T. gondii* strain was used because most cases of human toxoplasmosis are associated with *T. gondii* strains of genotype II [27]. The number of *T. gondii* cysts was counted using a light microscope and adjusted to 100 cysts/ml in PBS. Each cat in the infected group was orally infected with 100 cysts. Cats in the control group were given PBS orally only. On day 7 post-infection, the cats were euthanized by overdose of isoflurane. This procedure was performed by a qualified veterinarian with knowledge of anesthetic techniques. After confirmation of complete unresponsiveness to stimuli and permanent cessation of chest movement and heart rate, the tissue samples were harvested and immediately stored at –80 °C until processing.

### Confirmation of *T. gondii* infection

Total genomic DNA of each tissue sample was isolated by using TIANamp Genomic DNA kit (TianGen™, Beijing, China) according to manufacturer’s recommendations. The extracted DNA was used to detect the *T. gondii* infection by using a PCR assay that targets *T. gondii* B1 gene [28], The genotype of the detected *T. gondii* was determined by using PCR–RFLP as described previously [29].

### Protein extraction

Tissue samples were ground into powder in liquid nitrogen and were treated with Lysis buffer (7 mol/L urea, 2 mol/L thiourea, 4% CHAPS, 40 mmol/L tris–HCl, pH 8.5) containing 1 mmol/L phenylmethylsulfonyl fluoride (PMSF) and 2 mmol/L ethylenediaminetetraacetate (EDTA) for 5 min. Then, 10 mmol/L dithiothreitol (DTT) was added to the samples. The suspension was sonicated at 200 W for 15 min and centrifuged at 4 °C, 30,000 × *g* for 15 min. After mixing the supernatant with chilled acetone containing 10% (v/v) trichloroacetic acid, the mixture was stored at − 20 °C for overnight. The mixture was centrifuged at 4000 × *g* for 10 min at 4 °C. The pellet of the protein was washed three times with pre-cold acetone. The air-dried pellet was dissolved into Lysis buffer (7 mol/L urea, 2 mol/L thiourea, 4% NP40, 20 mmol/L Tris–HCl, pH 8.0 − 8.5) and was sonicated at 200 W for 15 min. The supernatant was collected into a new tube after centrifuging at 50,000 × *g* for 10 min at 4 °C. Then, 10 mmol/L DTT was added into the supernatant and the mixture was incubated for 1 h at 56 °C. The above mixture was placed in the dark for 1 h after adding 55 mmol/L iodoacetamide (IAM) to block free thiols and preserve cysteines. Then the mixture was added with chilled acetone again and was centrifuged at 4000 × *g* for 10 min at 4 °C to collect the pellet. After dissolving the pellet in 500 μl 0.5 mol/L triethylammonium bicarbonate (TEAB) (Applied Biosystems, Milan, Italy), the pellet was transferred to a new tube after discarding the supernatant. Finally, Bradford Protein Assay (Bio-Rad, Hercules, USA) was used to determine the protein concentration.

### iTRAQ labeling and SCX fractionation

Proteins (100 μg) per sample were digested using Trypsin Gold (Promega, Madison, WI, USA) at a 30:1 protein: trypsin ratio for 16 h at 37 ℃. After digestion, peptides were dried by vacuum centrifugation and were reconstituted in 0.5 mol/L TEAB and processed according to the manufacturer’s instructions for 8-plex iTRAQ reagent (Applied Biosystems, Calif, USA). Strong cation exchange (SCX) chromatography was conducted using LC-20AB HPLC Pump system (Shimadzu, Kyoto, Japan). The iTRAQ-labeled peptide mixtures were reconstituted with 4 ml buffer A (25 mm NaH_2_PO_4_ in 25% ACN, pH 2.7) and loaded onto a 4.6 × 250 mm Ultremex SCX column containing 5 μmol/L particles (Phenomenex, Calif, USA). The peptides were eluted at a flow rate of 1 ml/min with a gradient of buffer A for 10 min, 5–60% buffer B (25 mm NaH_2_PO_4_, 1 mol/L KCl in 25% ACN, pH 2.7) for 27 min, 60–100% buffer B for 1 min. The system was then maintained at 100% buffer B for 1 min before equilibrating with buffer A for 10 min prior to the next injection. Elution was monitored by measuring the absorbance at 214 nm, and fractions were collected every 1 min. The eluted peptides were pooled into 20 fractions, desalted with a Strata X C18 column (Phenomenex, Calif, USA) and vacuum dried.

### Liquid chromatography-tandem mass spectrometry (LC–MS/MS) analysis

Each fraction was re-suspended in buffer A (5% ACN, 0.1% FA) and centrifuged at 20,000 × *g* for 10 min, and the final concentration of peptide was 0.5 μg/μl. Approximately 10 μl supernatant was loaded on a LC-20AD nanoHPLC (Shimadzu, Kyoto, Japan) by an auto sampler onto a 2 cm C18 trap column. The peptides were then eluted onto a 10 cm analytical C18 column (inner diameter 75 μm) packed in-house. The samples were loaded at 8 μl/min for 4 min; then the 35 min gradient was run at 300 nl/min starting from 2 and increasing to 35% buffer B (95% ACN, 0.1% FA), followed by 5 min linear gradient to 60% and 2 min linear gradient to 80%, and maintenance at 80% buffer B for 4 min, and finally a return to 5% for 1 min.

Data acquisition was conducted with a TripleTOF 5600 System (AB SCIEX, Concord, ON) fitted with a Nanospray III source (AB SCIEX, Concord, ON) and a pulled quartz tip as the emitter (New Objectives, Woburn, MA). Data was acquired using an ion spray voltage of 2.5 kV, curtain gas of 30 psi, nebulizer gas of 15 psi, and an interface heater temperature of 150 °C. The MS was operated with a resolution power (RP) of ≥ 30,000 (full width at half maxima; FWHM) for TOF MS scans. For information dependent acquisition (IDA), the survey scans were acquired in 250 MS and as many as 30 product ion scans were collected if exceeding a threshold of 120 counts per second (counts/s) and with a 2 + to 5 + charge-state. Total cycle time was fixed to 3.3 s. Q2 transmission window was 100 Da for 100%. Four-time bins were summed for each scan at a pulser frequency value of 11 kHz via monitoring of the 40 GHz multichannel TDC detector with four-anode channel detect ion. A sweeping collision energy setting of 35 ± 5 eV coupled with iTRAQ adjust rolling collision energy was applied to all precursor ions for collision-induced dissociation. Dynamic exclusion was set for 1/2 of peak width (15 s), and then the precursor was refreshed off the exclusion list.

### Protein identification and data analysis

Mascot search engine (version 2.3.02, Matrix Science, London, UK) against the Uniprot database was used for protein identification. Mascot software was used to conduct the student’s *t*-test. The resulting dataset was auto bias-corrected to the biological replicates. Proteins with *P* values < 0.05 and fold change > 1.2 or < 0.83 were considered as differentially expressed proteins (DEPs). Web-based software (http://www.geneontology.org) was used to conduct gene ontology (GO) annotation analysis of the potential biological functions of significantly dysregulated proteins [30]. Kyoto Encyclopedia of Genes and Genomes database (KEGG, http://www.genome.p/kegg) was used to perform pathway analysis [31]. EuKaryotic Orthologous Groups (KOG, https://ftp.ncbi.nih.gov/pub/COG/KOG/) was used to detect the functional annotation of the DEPs. STRING 10.0 (http://string-db.org) was employed to explore the interaction network and functional relations of the DEPs.

### Correlation analysis between the proteome and transcriptome

In a previous study, parallel tissue samples were harvested from the same cat organs (brain, heart, liver, lung, small intestine and spleen), on day 7 after initial *T. gondii* infection. RNAs isolated from those samples were used in RNA sequencing to profile the transcriptomes of multiple cat organs [25]. The basic principle of the association analysis between proteome and transcriptome is the central dogma. The data screening and difference definitions of this association analysis were as follow: protein-fold change > 1.2, gene fold change > 2, gene significant < 0.001, GO and pathway significant < 0.05. Correlation was considered if a gene/protein was differentially expressed at both the proteome and transcriptome level after *T. gondii* infection. We also analyzed the level of concordance between transcripts and proteins of genes that belong to the same pathway. Pathways with good congruence between changes of the protein and transcript levels suggest that changes in the expression of transcripts may have changed the expression of the respective proteins; hence, modest alterations occur at the post-transcriptional regulation level.

## Results

### Confirmation of *T. gondii* infection

Positive PCR results were detected for the six organs obtained from infected cats (Additional file 1: Fig. S1A), and the positive PCR products were identified as *T. gondii* genotype II by RFLP analysis. As anticipated, all samples from control cats were negative (Additional file 1: Fig. S1A). *T. gondii* oocysts were observed in the feces of infected cats (Additional file 1: Fig. S1B).

### The identification and quantification of proteins

We used iTRAQ-based quantitative proteomic approach to identify the protein species and their expression levels in different tissue samples of acutely infected cats. A total of 2,118,641 spectra were obtained, with an average of 353,107 spectra per organ. According to the Mascot program searching, an average of 78,698 spectra were matched and an average of 68,794 high scoring unique peptides were obtained. 32,657 proteins were identified in the six organs, with an average of 5443 proteins per organ (Table 1). For differential expression analysis, 855, 575, 463, 283, 255, and 125 proteins were identified as DEPs in the heart, small intestine, liver, spleen, lung, and brain, respectively, compared to non-infected groups (Table 2). Of the 2556 DEPs, 1325 and 1231 were identified as up- and down-regulated proteins in infected vs uninfected cats. Liver had the largest number of up-regulated proteins (319), followed by small intestine (309), heart (298), spleen (185), lung (155), while the lowest number was detected in the brain (59). Interestingly, the largest number of down-regulated proteins was detected in the heart (557), followed by small intestine (266), liver (144), lung (100), spleen, while the lowest number was detected in the brain (66). The numbers of the DEPs of each organ are listed in Table 2.

**Table 1:** Summary of the proteomics results

Organ	Total spectra	Spectra	Unique spectra	Peptide	Unique peptide	Protein
Brain	398,525	87,146	75,783	33,954	31,331	6127
Heart	308,981	74,694	65,857	30,514	28,626	5824
Spleen	296,239	62,826	53,535	21,352	19,661	4196
Liver	355,179	79,050	72,856	27,349	25,923	5180
Lung	379,722	84,857	72,555	27,541	25,457	5435
Small intestine	379,995	83,614	72,178	30,669	28,606	5895

**Table 2:** Summary of the identified proteins in the cat organs

Organ	Total proteins	DEPs	UP-regulated	Down-regulated
Brain	6127	125	59	66
Liver	5180	463	319	144
Lung	5435	255	155	100
Spleen	4196	283	185	98
Heart	5824	855	298	557
Small intestine	5895	575	309	266
Total	32,657	2556	1325	1231

*DEPs* differentially expressed proteins

### Functional enrichment analysis of all proteins

**Fig. 1:**
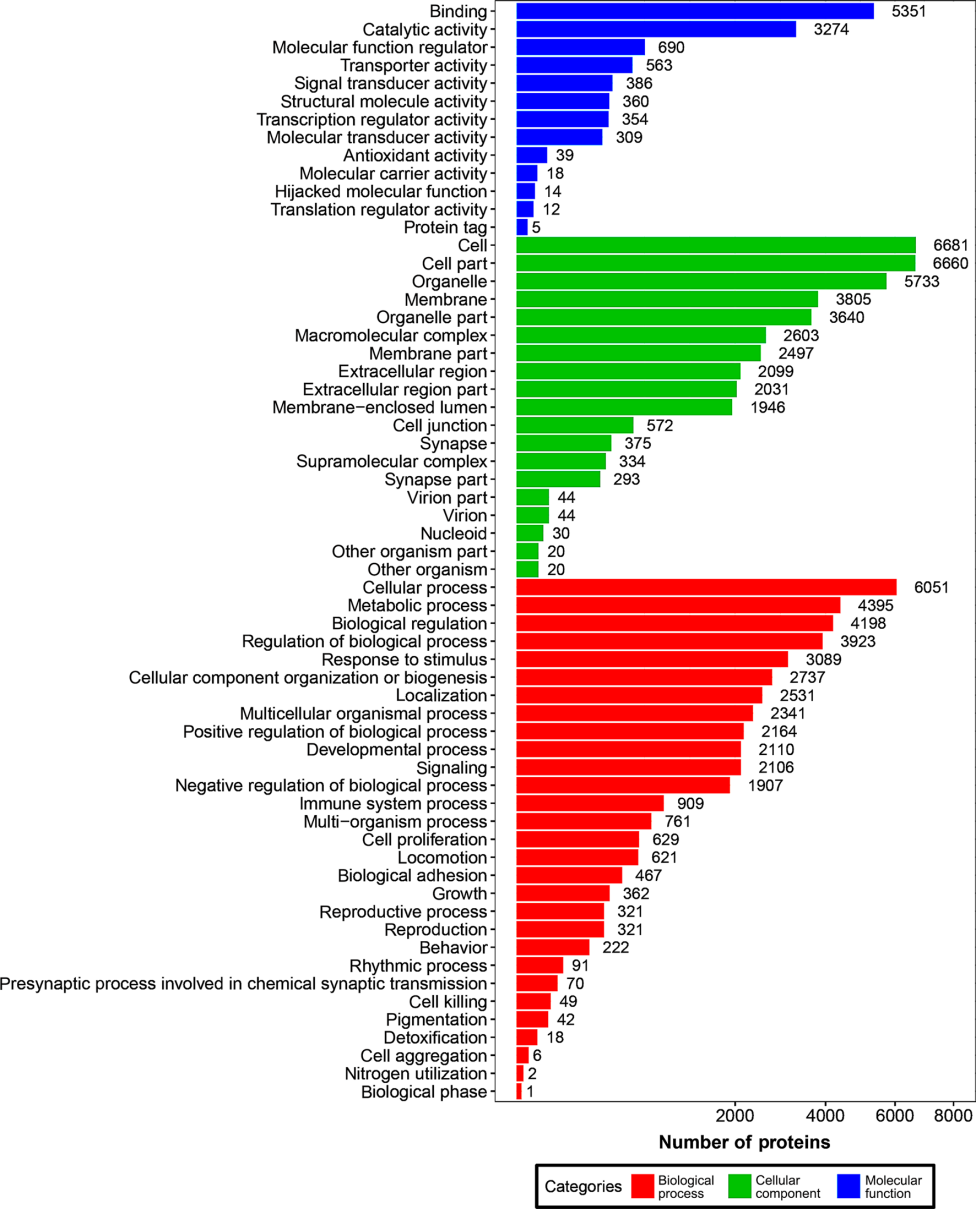
Horizontal bar graph of gene ontology (GO) classification of proteins identified in the brain, liver, lung, spleen, heart and small intestine of cats infected by *Toxoplasma gondii* on day 7 post-infection. X-axis shows the number of proteins and y-axis shows the GO terms

**Fig. 2:**
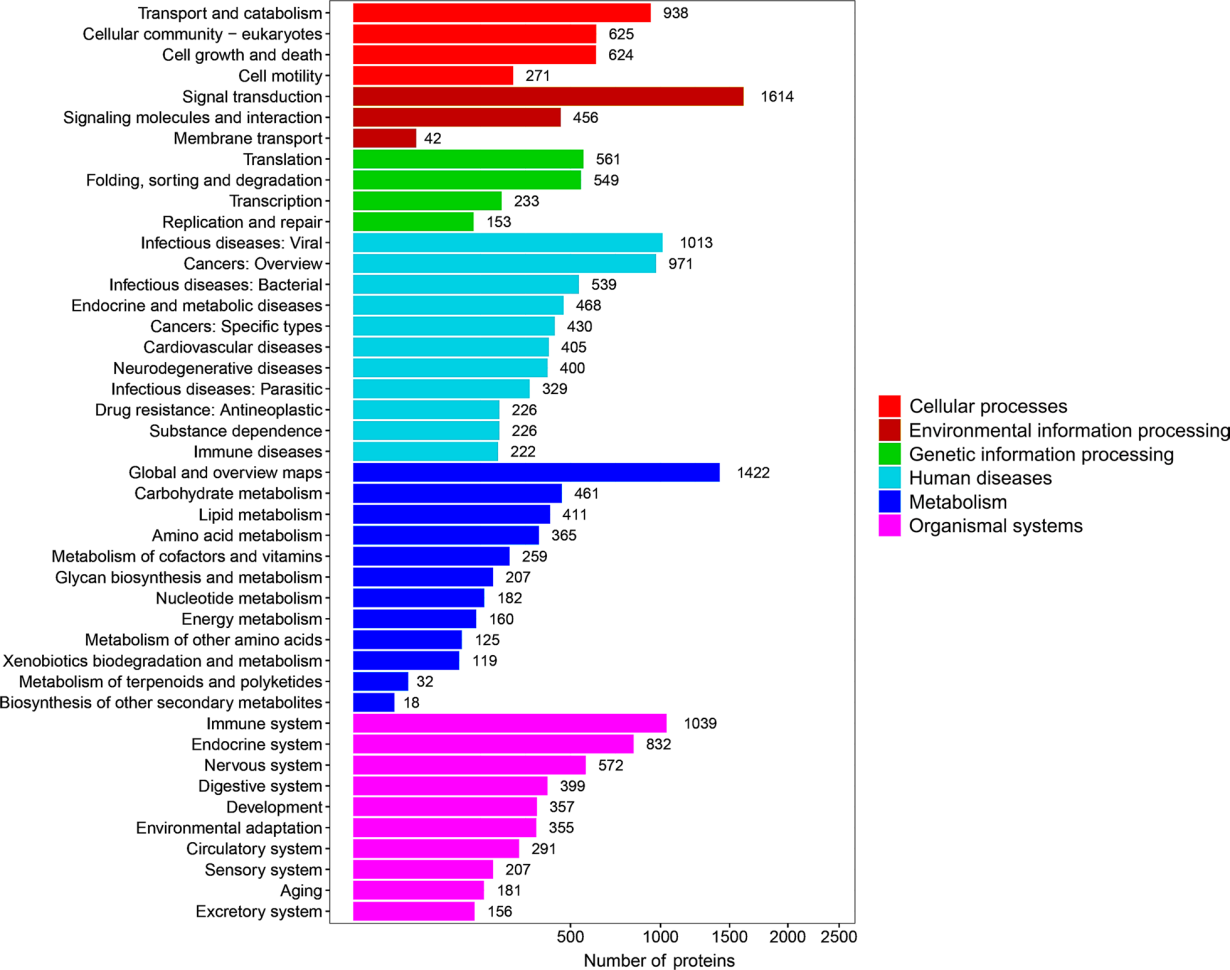
Kyoto Encyclopedia of Genes and Genomes (KEGG) pathway enrichment of the proteins identified in the brain, liver, lung, spleen, heart and small intestine of cats infected by *Toxoplasma gondii* on day 7 post-infection. X-axis shows the number of proteins and y-axis shows the KEGG pathways

GO and KEGG pathway enrichment analyses were performed on all proteins and the detailed results are shown in Additional file 2: Table S1 and Additional file 3: Table S2. Based on the GO enrichment results of the biological process (BP) category, 5351 and 3274 proteins were significantly enriched in binding and catalytic activity. Proteins were annotated into different cellular components (CC) category, including cell body (13,341), organelle (9373), membrane (6302), and macromolecular complex (2603). For the molecular function (MF) category, 6051, 4395, 4198 and 3923 proteins were associated with cellular process, metabolic process, biological process and regulation of biological process, respectively (Fig. 1). KEGG enrichment identified six functional categories, including cellular processes, environmental information processing, genetic information process, human diseases, metabolism, and organismal system. Most proteins were enriched in transport and catabolism (938), signal transduction (1614), translation (561), infectious diseases: viral (1013), global and overview maps (1422), and immune system (1039) (Fig. 2).

### Enrichment analysis of differentially expressed proteins

In the MF category, most DEPs were enriched in binding, catalytic activity, molecular function regulator, structural molecule activity and transporter activity (Table 3). For CC category, most DEPs were enriched in cell part, cell, organelle, extracellular region, membrane, and macromolecular complex (Table 3). In the BP category, most DEPs were involved in cellular process, metabolic process, biological regulation, regulation of biological process, and response to stimulus. The DEPs in brain were enriched in multicellular organismal process (Table 3).

**Table 3:** Summary of GO enrichment analysis of the cat organs

Organ	Molecular function	Cellular component	Biological process
Brain	Binding; catalytic activity; transporter activity; molecular function regulator; signal transducer activity	Cell part; cell; organelle; membrane; macromolecular complex	Regulation of biological process; biological regulation; cellular process; multicellular organismal process; metabolic process
Heart	Binding; catalytic activity; transporter activity; molecular function regulator; structural molecule activity	Cell part; cell; organelle; membrane; macromolecular complex	Cellular process; metabolic process; biological regulation; regulation of biological process; response to stimulus
Spleen	Binding; catalytic activity; structural molecule activity; molecular function regulator; transporter activity	Cell part; cell; organelle; membrane; macromolecular complex	Cellular process; metabolic process; biological regulation; regulation of biological process; response to stimulus
Liver	Binding; catalytic activity; structural molecule activity; molecular function regulator; transporter activity	Cell part; cell; organelle; extracellular region; extracellular region part	Cellular process; metabolic process; biological regulation; regulation of biological process; response to stimulus
Lung	Binding; catalytic activity; molecular function regulator; structural molecule activity; signal transducer activity	Cell part; cell; organelle; extracellular region; extracellular region part	Cellular process; metabolic process; biological regulation; regulation of biological process; response to stimulus
Small intestine	Binding; catalytic activity; molecular function regulator; transporter activity; structural molecule activity	Cell part; cell; organelle; extracellular region; extracellular region part	Cellular process; metabolic process; biological regulation; regulation of biological process; response to stimulus

*GO* Gene Ontology

KOG enrichment analysis showed that most DEPs were enriched in amino acid transport and metabolism (small intestine, lung), energy production and conversion (spleen, heart), lipid transport and metabolism (liver, brain) in metabolism. In information storage and processing, most DEPs were enriched in translation, ribosomal structure and biogenesis (small intestine, lung, spleen), RNA processing and modification (liver), chromatin structure and dynamics replication (heart), replication, recombination and repair (brain). In cellular processes and signaling, the DEPs were mainly associated with signal transduction mechanisms (brain, heart, spleen, liver, lung) and posttranslational modification, protein turnover, chaperones (small intestine) (Table 4).

**Table 4:** Summary of KOG enrichment analysis of the cat organs

Organ	Poorly characterized	Metabolism	Information storage and processing	Cellular processes and signaling
Brain	General function prediction only	Lipid transport and metabolism	Replication, recombination and repair	Signal transduction mechanisms
Heart	General function prediction only	Energy production and conversion	Chromatin structure and dynamics	Signal transduction mechanisms
Spleen	General function prediction only	Energy production and conversion	Translation, ribosomal structure and biogenesis	Signal transduction mechanisms
Liver	General function prediction only	Lipid transport and metabolism	RNA processing and modification	Signal transduction mechanisms
Lung	General function prediction only	Amino acid transport and metabolism	Translation, ribosomal structure and biogenesis	Signal transduction mechanisms
Small intestine	General function prediction only	Amino acid transport and metabolism	Translation, ribosomal structure and biogenesis	Posttranslational modification, protein turnover, chaperones

*KOG* EuKaryotic Orthologous Groups

As shown in Table 5, KEGG pathway enrichment revealed that in cellular processes, most DEPs were associated with cell growth and death (small intestine, spleen, brain) or transport and catabolism (lung, liver, heart). In environmental information processing, most DEPs were associated with signal transduction and translation (small intestine, liver,spleen, lung) or folding, sorting and degradation (brain, heart). In genetic information processing, most DEPs were associated with folding, sorting and degradation (brain, heart) or translation (spleen, liver, lung, small intestine). In human diseases, most DEPs were associated with cancers: overview (small intestine) or infectious diseases (lung, liver): viral or cardiovascular diseases (spleen) or neurodegenerative diseases (heart) or cardiovascular disease (brain). In metabolism, most DEPs were associated with global and overview maps. In organismal systems, most DEPs were associated with immune system (brain, spleen, liver, lung, small intestine) or environmental adaption (heart).

**Table 5:** Summary of the pathway enrichment analysis of the cat organs showing enriched pathways annotated according to the six main KEGG categories

Organ	Cellular processes	Environmental information processing	Genetic information processing	Human diseases	Metabolism	Organismal systems
Brain	Cell growth and death	Signal transduction	Folding, sorting and degradation	Cardiovascular diseases	Global and overview maps	Immune system
Heart	Transport and catabolism	Signal transduction	Folding, sorting and degradation	Neurodegenerative diseases	Global and overview maps	Environmental adaptation
Spleen	Cell growth and death	Signal transduction	Translation	Cardiovascular diseases	Global and overview maps	Immune system
Liver	Transport and catabolism	Signal transduction	Translation	Infectious diseases: Viral	Global and overview maps	Immune system
Lung	Transport and catabolism	Signal transduction	Translation	Infectious diseases: Viral	Global and overview maps	Immune system
Small intestine	Cell growth and death	Signal transduction	Translation	Cancers: Overview	Global and overview maps	Immune system

*KEGG* Kyoto Encyclopedia of Genes and Genomes

### Correlations between the proteome and transcriptome data

**Fig. 3:**
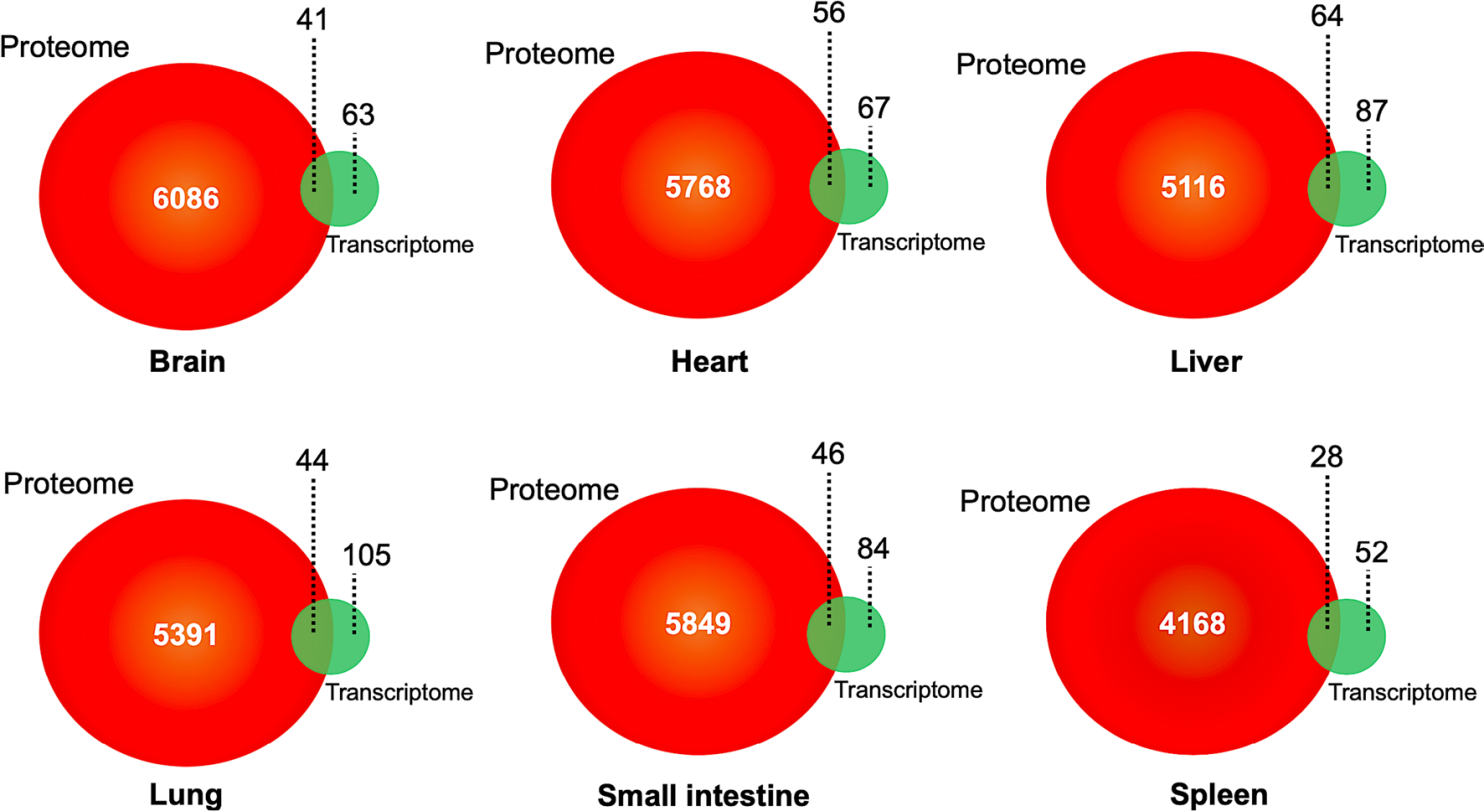
Venn diagrams showing overlap between all proteins identified in the proteome and differentially expressed genes identified in the transcriptome of the brain, heart, liver, lung, small intestine and spleen of cats infected by *Toxoplasma gondii* on day 7 post-infection

**Fig. 4:**
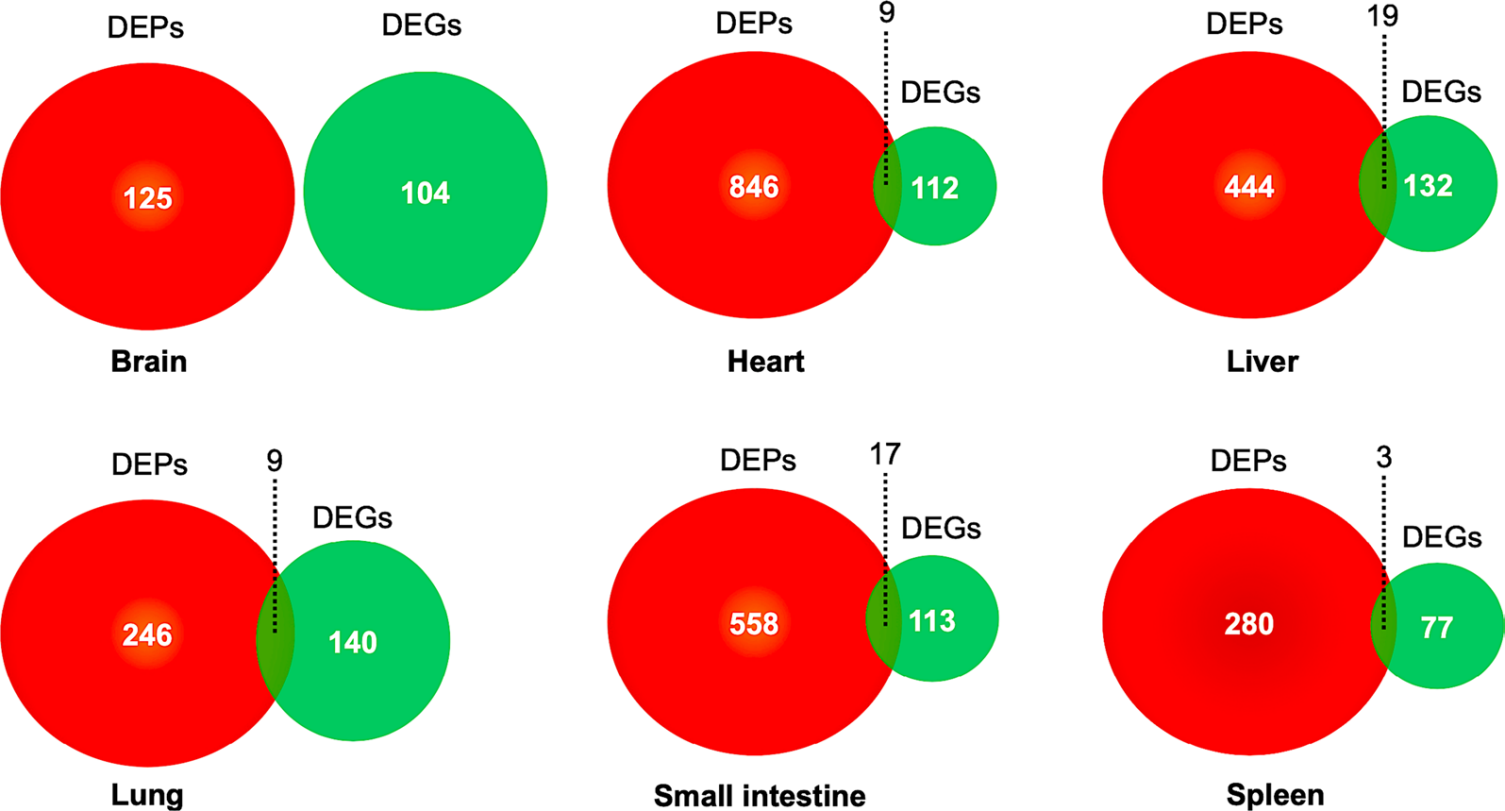
Venn diagrams showing overlap between differentially expressed proteins (DEPs) identified in the proteome and differentially expressed genes (DEGs) identified in the transcriptome of the brain, heart, liver, lung, small intestine and spleen of cats infected by *Toxoplasma gondii* on day 7 post-infection

A low correlation between the level of transcript and protein changes was observed for all quantified proteins (*R* = 0.027) and differentially expressed proteins (*R* = 0.487). In all proteins, 41, 56, 64, 44, 46, 28 proteins were commonly identified at the protein and transcript levels in the brain, heart, liver, lung, small intestine, and spleen, respectively (Fig. 3). While for the DEPs, only 19, 17, 9, 9, 3 proteins were common at the transcript and protein levels in the liver, small intestine, heart, lung, and spleen (Fig. 4; Additional file 4: Table S3). Among those, 3 proteins (indoleamine 2, 3-dioxygenase 1 and guanylate binding protein 1), 12 proteins (e.g. indoleamine 2, 3-dioxygenase 1, caveolae associated protein, parvin beta), 8 proteins (e.g. indoleamine 2, 3-dioxygenase 1, synapsin I, matrix metallopeptidase), 8 proteins (e.g. indoleamine 2, 3-dioxygenase 1, tryptophanyl-tRNA synthetase, apolipoprotein A1) and 3 proteins (transmembrane p24 trafficking protein 1, CDP-diacylglycerol–inositol 3-phosphatidyltransferase, importin subunit alpha) were upregulated in the heart, liver, lung, small intestine and spleen, respectively. Seven proteins (e.g. collagen type XV alpha 1, collagen type I alpha 1, FTO alpha-ketoglutarate dependent dioxygenase), 7 proteins (e.g. glucosylceramidase beta 3, NDRG family member 2, carboxylic ester hydrolase), 1 protein (reticulon) and 9 proteins (e.g. UDP-glucuronosyltransferase, ectonucleotide pyrophosphatase/phosphodiesterase 6, 3-hydroxyanthranilate 3, 4-dioxygenase) were downregulated in the heart, liver, lung and small intestine, respectively (Additional file 4: Table S3). Interestingly, there were no common DEPs between the proteome and transcriptome in the brain (Fig. 4).

**Fig. 5:**
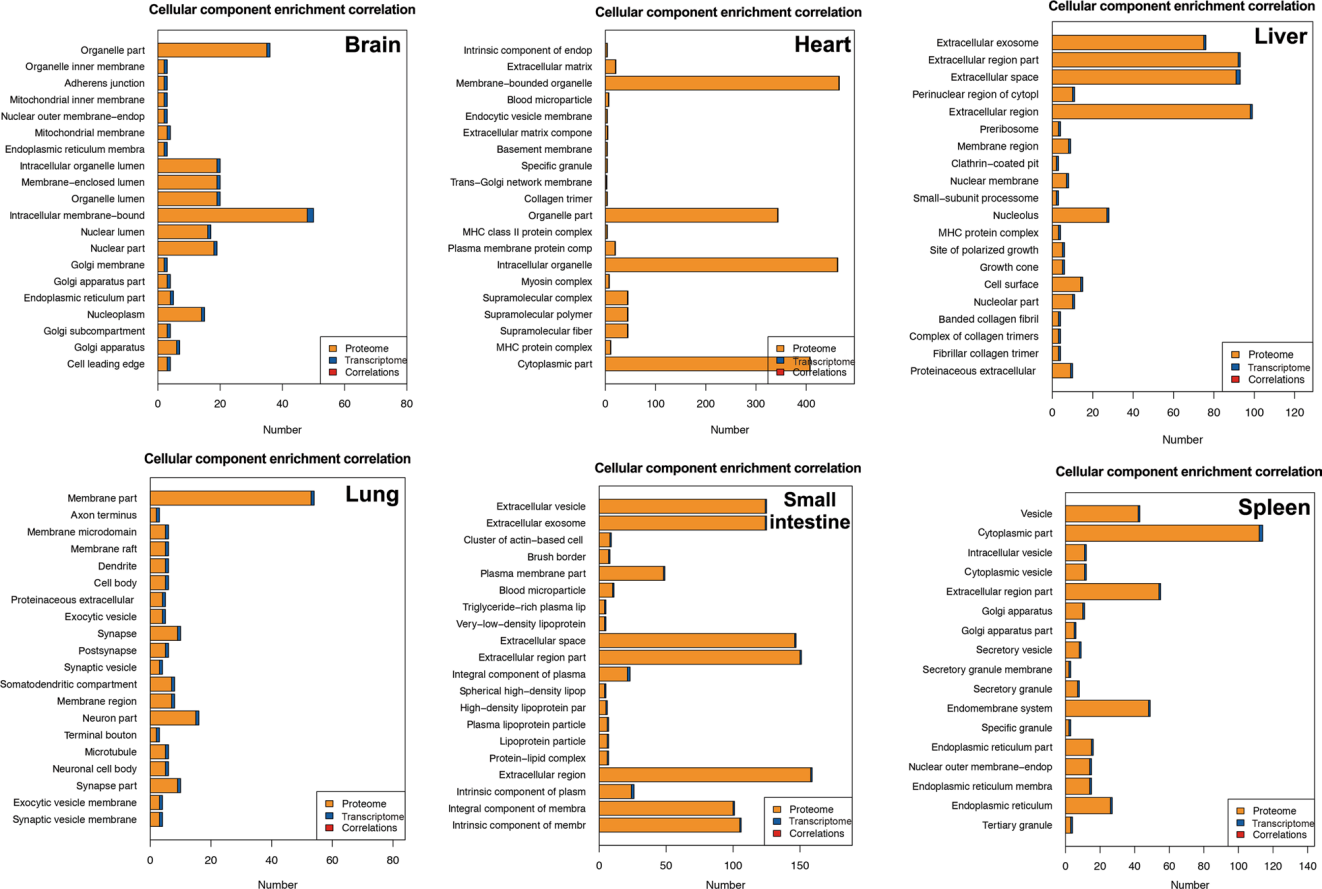
Gene Ontology (GO)-correlation enrichment of the differentially expressed proteins (DEPs) in the cellular component showing the proportion of changes at the protein and transcript levels in the brain, heart, liver, lung, small intestine and spleen of cats infected by *Toxoplasma gondii* on day 7 post-infection

**Fig. 6:**
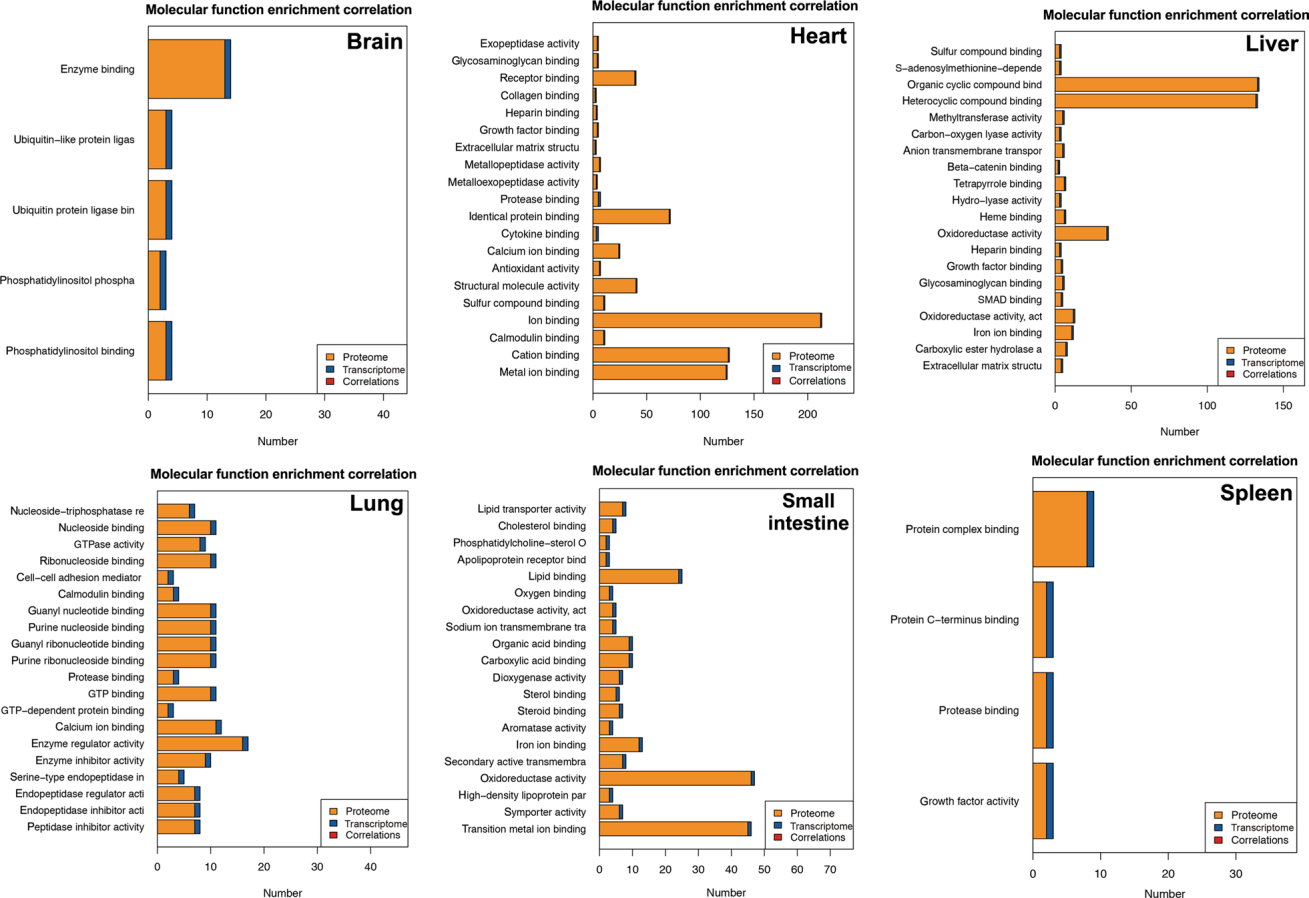
Gene Ontology (GO)-correlation enrichment of the differentially expressed proteins (DEPs) in the molecular function showing the proportion of changes at the protein and transcript levels in the brain, heart, liver, lung, small intestine and spleen of cats infected by *Toxoplasma gondii* on day 7 post-infection

**Fig. 7:**
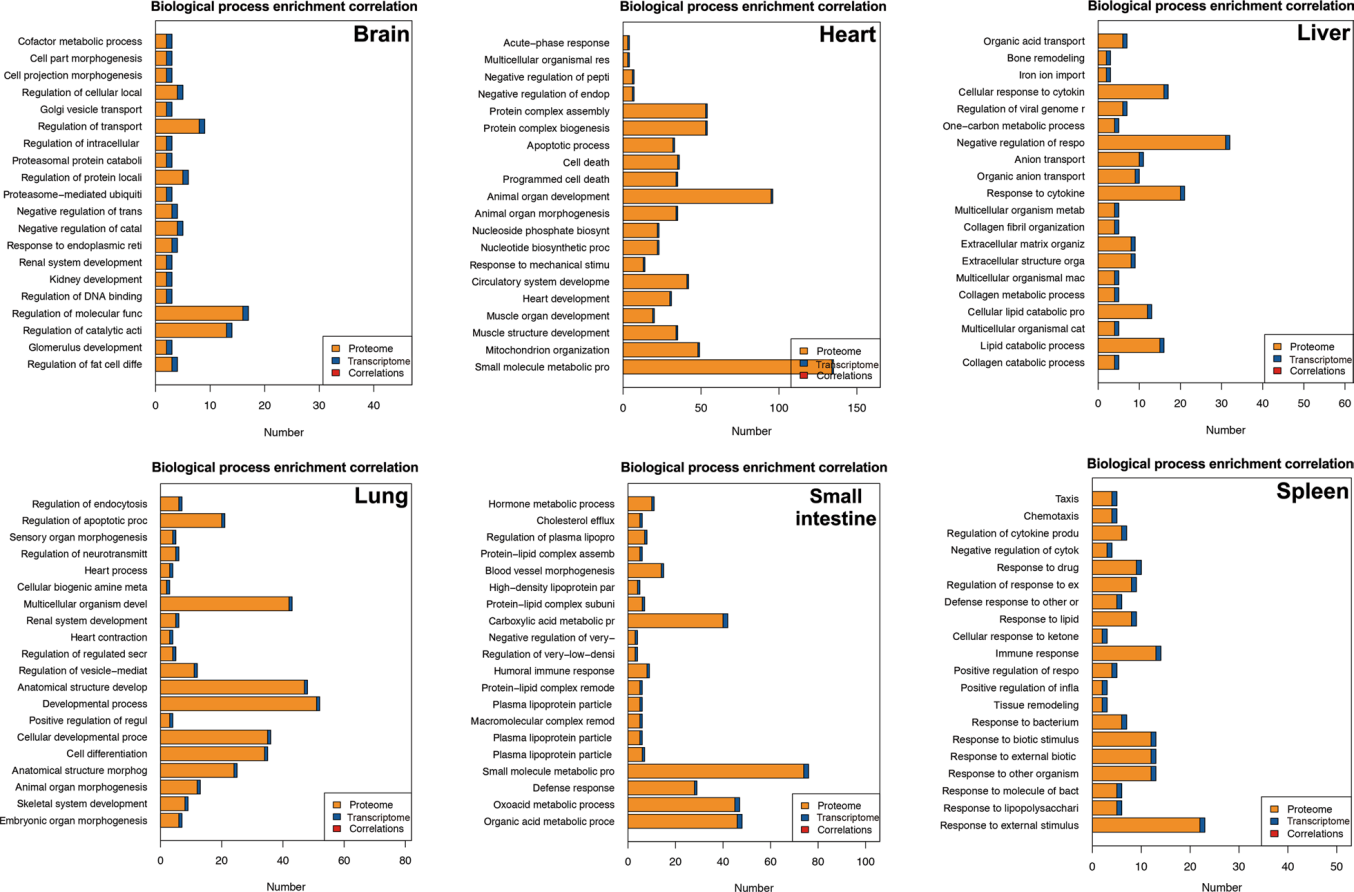
Gene Ontology (GO)-correlation enrichment of the differentially expressed proteins (DEPs) in the biological process showing the proportion of changes at the protein and transcript levels in the brain, heart, liver, lung, small intestine and spleen of cats infected by *Toxoplasma gondii* on day 7 post-infection

**Fig. 8:**
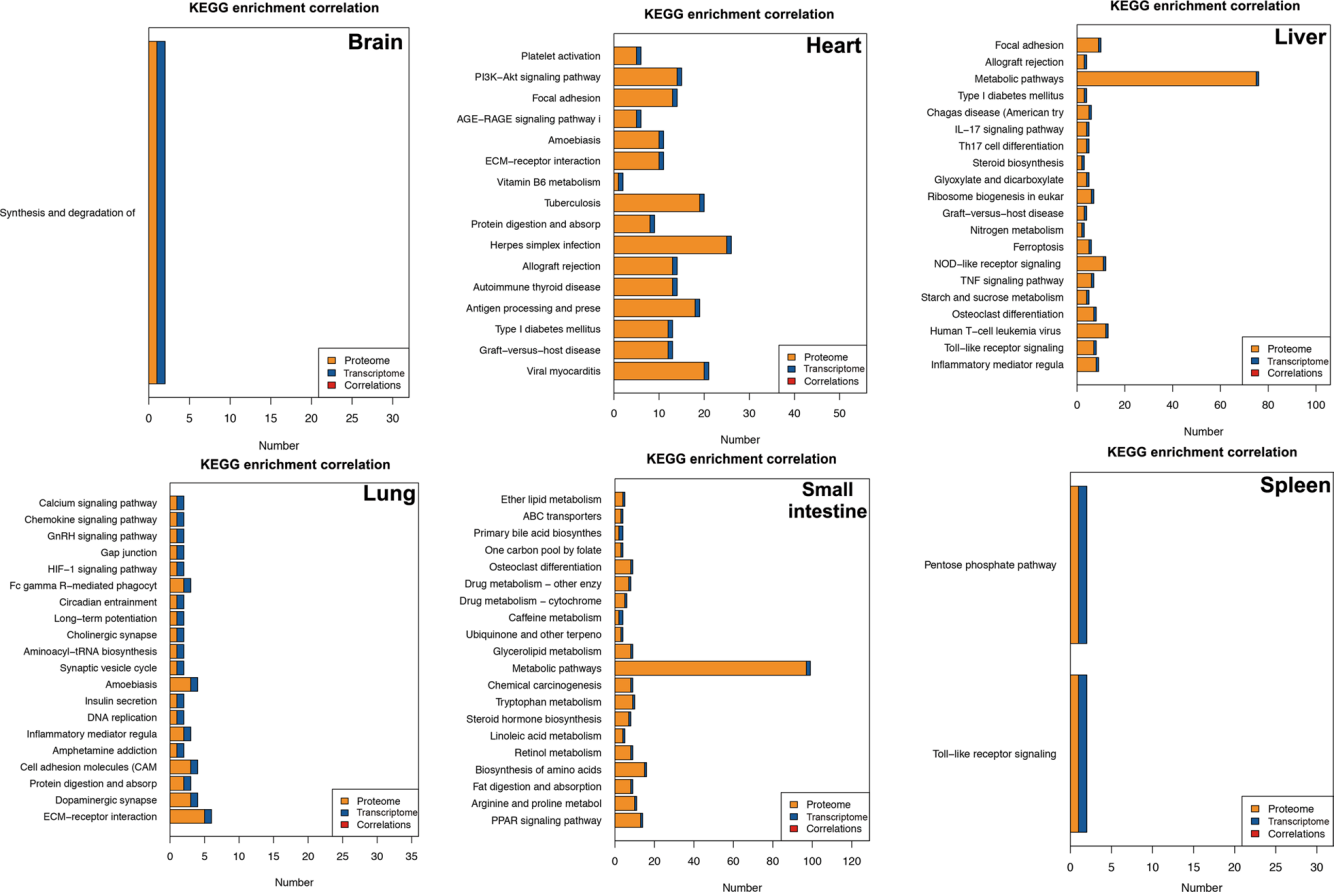
Kyoto Encyclopedia of Genes and Genomes (KEGG)-correlation enrichment of the differentially expressed proteins (DEPs) showing the proportion of changes at the protein and transcript levels in the brain, heart, liver, lung, small intestine and spleen of cats infected by *Toxoplasma gondii* on day 7 post-infection

For the GO-correlation analysis, the DEPs were mainly enriched in intracellular membrane-bound, extracellular region, membrane part, and cytoplasmic part in the CC category (Fig. 5). In the MF category, most DEPs were enriched in enzyme binding, ion binding, organic cyclic compound bind, enzyme regulator activity, oxidoreductase activity, and protein complex binding (Fig. 6). In the BP category, most DEPs were enriched in the regulation of molecular function, small molecule metabolic process, negative regulation of response, developmental process, and response to external stimulus (Fig. 7). KEGG pathway enrichment correlation revealed that DEPs are mainly enriched in herpes simplex infection, metabolic pathway, ECM-receptor interaction, Toll-like receptor signaling, and pentose phosphate pathway (Fig. 8).

## Discussion

Cats play a fundamental role in the biology and epidemiology of *T. gondii* because they are the only definitive hosts that can produce and excrete *T. gondii* oocysts. In a previous study, we determined the transcriptomic profiles of the main cat organs (brain, heart, spleen, liver, lung and small intestine) at 7 days post infection by *T. gondii* [25]. Aiming for a more comprehensive picture of the molecular response of cat tissues to *T. gondii* infection, we studied the global differential protein expression profile of the same cat tissues and at the same time point post infection using iTRAQ-based quantitative proteomics.

A total of 2556 DEPs were identified in all cat organs; of which 1325 and 1231 were identified as up-regulated and down-regulated proteins, respectively. The proteomic profiling also showed that 319, 309 and 298 DEPs were up-regulated in the liver, small intestine and heart, while 557 and 266 DEPs were down-regulated in the heart and small intestine, respectively. These results show that liver had the largest up-regulated proteins (*n* = 319), while the heart had the largest down-regulated proteins (*n* = 557). This corroborates part of the results in a previous transcriptomic study where the liver had the highest number of differentially expressed transcripts, suggesting that *T. gondii* causes significant changes in the liver [25]. The results also suggest that heart had the highest level of DEPs (298 up-regulated + 557 down-regulated = 855 DEPs), indicating a significant impact of *T. gondii* on protein regulation in the heart compared to other organs. One would expect the heart to exhibit significant changes in proteins owing to the contractile function of the organ which requires a lot of metabolism and bioenergetics as was evident by the KOG enrichment of DEPs in energy production and conversion (Table 4). The difference in the differentially regulated transcripts and proteins between different tissues may suggest tissue-specific variations in the induction and turnover rates of transcripts and proteins.

The functional enrichment of the DEPs in the six cat tissues identified some important up-regulated proteins in heart, such as MHC 1, MHC II, heat shock protein, phosphoinositide phospholipase C (TgPI-PLC). MHC 1 plays a key role in processing and presenting *T. gondii* proteins to CD8^+^ T-cells [32]. Expression of MHC II by microglia and astrocytes is crucial for CD4^+^-mediated immune responses in the central nervous system [33]. These proteomic results concur with previous transcriptomic analysis showing *T. gondii*’s ability to induce significant expression of immune related genes in all infected organs in cats [25]. Heat shock protein can play key roles in the pathogenesis of *T. gondii* [34]. TgPI-PLC is localized in the internal leaflet of the plasma membrane and encodes a functional PI-PLC in *T. gondii* genome [35]. Some of the up-regulated proteins remain uncharacterized, and further studies should be carried out to elucidate their biological functions in the context of *T. gondii* infection.

Enrichment analysis of the identified proteins suggest that *T. gondii* alters the xenobiotic metabolism. Oxidoreductase activity was enriched and up-regulated in the heart, liver and small intestine, suggesting that potential involvement in drug metabolism in the liver and that targeting oxidoreductase may be part of a mechanism by which *T. gondii* interferes with drug pharmacokinetic [36]. This result disagrees with a previous study showing downregulation of oxidoreductase activity protein in mouse liver [37]. However, this difference may be attributed to different experimental methods or host specificity. In the present study, feline aminopeptidase was downregulated in small intestine. A previous study showed that feline aminopeptidase N serves as an entry receptor for viruses [38]. Whether feline aminopeptidase N serves as a functional receptor for *T. gondii* entry into the small intestine remains to be investigated.

We further examined the transcriptome and proteome data sets to identify differences in the correlation between proteins and transcripts with differential expression patterns in different tissues in response to *T. gondii* infection. Correlations were detected between proteins and the corresponding transcripts in the liver (*n* = 19), small intestine (*n* = 17), heart (*n* = 9), lung (*n* = 9), with spleen showing the least transcript–protein correlation (*n* = 3). Detailed information is available as supplemental material (see Additional file 4: Table S3). Overall, this result shows a limited accordance between alterations in proteins and transcripts, suggesting that protein synthesis, protein stability, post-transcriptional modifications may have played a role in determining the protein levels beyond transcript levels between different tissues [39].

It is noteworthy that the correlation between the differential expression pattens of indoleamine 2,3-dioxygenase at both protein and transcript levels was detected in all organs, with the exception of spleen. IDO was co-expressed in 5 feline tissues (brain, heart, liver, small intestine, and spleen) in the previous transcriptomic analysis [25]. Induction of IDO, mediated by interferon gamma (IFN-γ), plays a key role in degradation of the essential amino acid tryptophan, inhibiting the growth of *T. gondii* [40], and limiting neuroinflammation [41–43]. These findings largely corroborate previous studies linking IDO to various mechanisms that mediate the pathophysiology of *T. gondii* infection [36, 37, 44–48].

Matrix metallopeptidases (MMPs) are proteolytic enzymes that degrade extracellular matrix proteins such as collagen, fibronectin and laminin. In the present study, matrix metallopeptidase was upregulated at both the transcript and protein levels in *T. gondii* infected lungs compared with the lungs of uninfected cats (Additional file 4: Table S3). Interestingly, downregulation of protein and transcript of collagen type I alpha 1 and collagen type XV alpha 1 was detected in the heart. A previous study showed that matrix metalloproteinase (MMP)-2 and MMP-9 cleave fibronectin and induce astroglia reaction and leukocyte migration to the sites of *T. gondii* infection in the brain [49]. Several molecules involved in the immune and inflammatory responses during toxoplasmosis, such as interleukin (IL)-1, IL-23 and tumor necrosis factor alpha (TNFα), can increase MMP production in the brain [50]. Additionally, MMP-8, MMP-10 and tissue inhibitor of metalloproteinases-1 (TIMP-1) have important functions in regulating the perivascular accumulation and influx of lymphocytes into the brain to prevent the reactivation of dormant *T. gondii* cysts [51]. These results suggest that regulation of extracellular matrix and collagen may be involved in the immune response of cats during early *T. gondii* infection.

Understanding the pathogenic mechanisms of *T. gondii* infection in cats is critical for the development of intervention technologies against *T. gondii* infection because cats play a key role in the transmission of *T. gondii*. The identification of 2556 DEPs in six cat tissues responsive to *T. gondii* infection provides new and valuable resources for further exploration of the intricate mechanism of toxoplasmosis pathogenesis in cats.

## Conclusions

iTRAQ-based quantitative proteomic analysis identified 32,657 proteins; of those 2556 were DEPs in six cat tissues at day 7 after infection by *T. gondii* PRU strain. The proteins were not expressed at similar levels across cat tissues and the correlation of transcript/protein ratios across tissues was very low. Functional annotation and enrichment analyses revealed that the DEPs were localized in different cellular compartments and were associated with various cellular and metabolic processes, such as immune response, drug metabolism, tryptophan catabolism, extracellular matrix remodeling, and information storage and processing. These proteomic profiles serve as a rich foundation for future investigations of the intricate mechanisms that allow *T. gondii* to adapt and survive in different feline tissues.

## Supplementary Information

**Additional file 1: Fig. S1.** Confirmation of *Toxoplasma gondii* infection. **A** Agarose gel electrophoresis of PCR amplicons obtained by amplification of *T. gondii* B1 gene-specific fragment (96-bp) from cat tissue DNA. Lane M, DNA ladder and the numbers to the left refer to the size of DNA marker fragments; Lane 1, positive control; Lane 2, negative control without DNA template; Lanes 3–8: positive PCR products from brain, heart, liver, lung, spleen and small intestine of infected cats; Lanes 9–14: negative results of samples obtained from the equivalent tissues of the uninfected cats. **B** Sporulated oocysts were observed in the feces of infected cats.

**Additional file 2: Table S1.** The GO enrichment of all proteins in the brain, liver, lung, spleen, heart and small intestine of cats infected by *Toxoplasma gondii* on day 7 post-infection.

**Additional file 3: Table S2.** The KEGG enrichment of all proteins in the brain, liver, lung, spleen, heart and small intestine of cats infected by *Toxoplasma gondii* on day 7 post-infection.

**Additional file 4: Table S3.** The common differentially expressed proteins and transcripts in the brain, liver, lung, spleen, heart and small intestine of cats infected by *Toxoplasma gondii* on day 7 post-infection.

## Data Availability

All the mass spectrometry data have been submitted to the ProteomeXchange Consortium with the identifier PXD033778.

## Abbreviations

iTRAQ: Isobaric tags for relative and absolute quantification
PMSF: Phenylmethylsulfonyl fluoride
EDTA: Ethylenediaminetetraacetate
DTT: Dithiothreitol
IAM: Iodoacetamide
TEAB: Triethylammonium bicarbonate
IDA: Information dependent acquisition
DEPs: Differentially expressed proteins
GO: Gene Ontology
KEGG: Kyoto Encyclopedia of Genes and Genomes
KOG: EuKaryotic Orthologous Groups
SCX: Strong cation exchange
LC–MS/MS: Liquid chromatography-tandem mass spectrometry
RP: Resolution power
FWHM: Full width at half maxima
