# Supplementary figures and images for: Global proteomic profiling of multiple organs of cats (Felis catus) and proteome-transcriptome correlation during acute Toxoplasma gondii infection
Source: Infect Dis Poverty. 2022 Sep 14;11:96. doi: 10.1186/s40249-022-01022-7 (PMC9473462; doi:10.1186/s40249-022-01022-7)

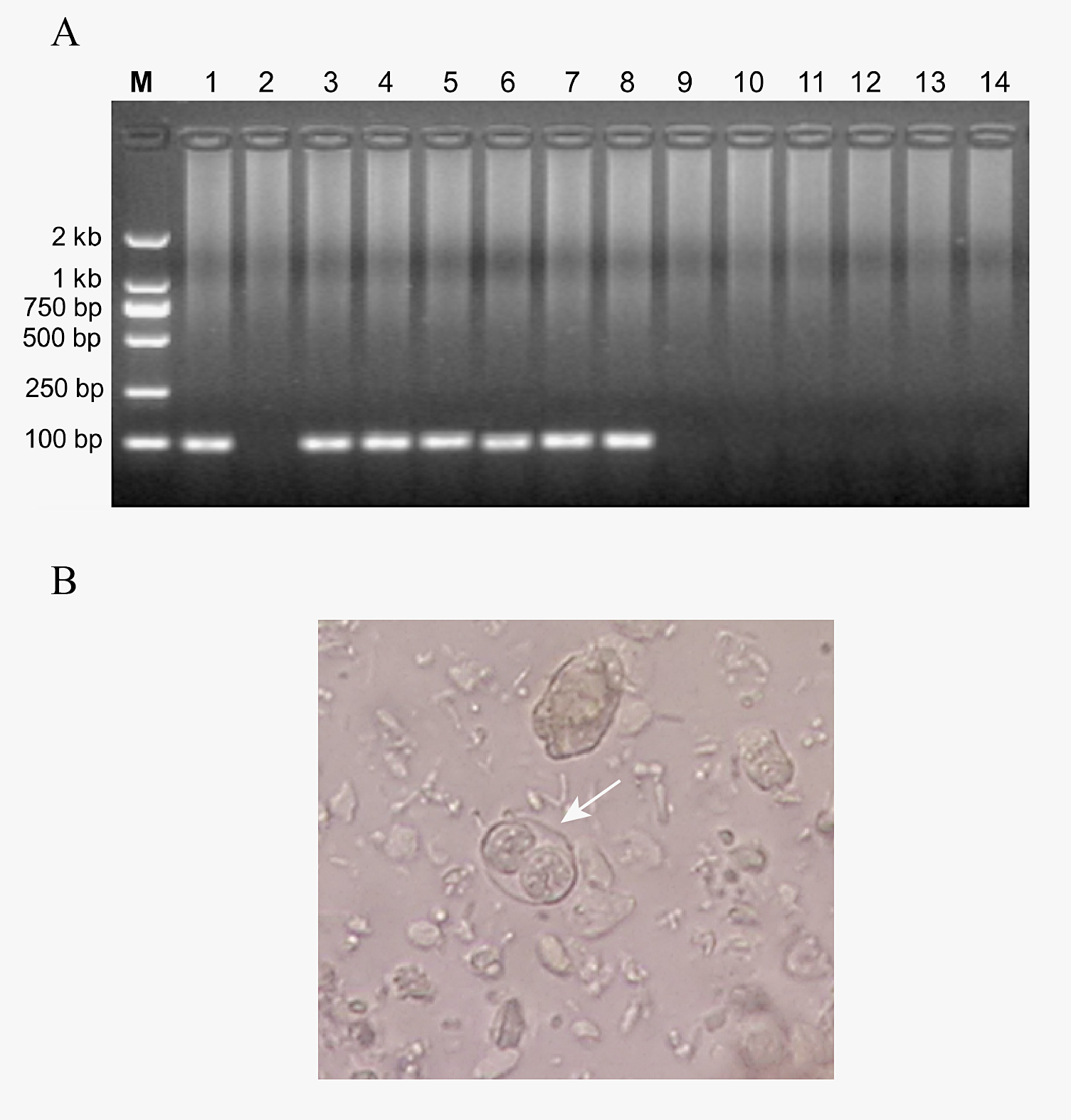

Supplement: Supplementary file 1 — Additional file 1: Fig. S1. Confirmation of Toxoplasma gondii infection. A Agarose gel electrophoresis of PCR amplicons obtained by amplification of T. gondii B1 gene-specific fragment (96-bp) from cat tissue DNA. Lane M, DNA ladder and the numbers to the left refer to the size of DNA marker fragments; Lane 1, positive control; Lane 2, negative control without DNA template; Lanes 3–8: positive PCR products from brain, heart, liver, lung, spleen and small intestine of infected cats; Lanes 9–14: negative results of samples obtained from the equivalent tissues of the uninfected cats. B Sporulated oocysts were observed in the feces of infected cats. [file 40249_2022_1022_MOESM1_ESM.tiff]
